# Application and visualization study of an intelligence-assisted classification model for common eye diseases using B-mode ultrasound images

**DOI:** 10.3389/fnins.2024.1339075

**Published:** 2024-05-14

**Authors:** Shaojun Zhu, Xiangjun Liu, Ying Lu, Bo Zheng, Maonian Wu, Xue Yao, Weihua Yang, Yan Gong

**Affiliations:** ^1^School of Information Engineering, Huzhou University, Huzhou, China; ^2^Shenzhen Eye Institute, Shenzhen Eye Hospital, Jinan University, Shenzhen, China; ^3^Department of Ophthalmology, Ningbo Eye Hospital, Wenzhou Medical University, Ningbo, China

**Keywords:** B-mode ultrasonography, common eye diseases, deep learning, visualization, classification, image

## Abstract

**Aim:**

Conventional approaches to diagnosing common eye diseases using B-mode ultrasonography are labor-intensive and time-consuming, must requiring expert intervention for accuracy. This study aims to address these challenges by proposing an intelligence-assisted analysis five-classification model for diagnosing common eye diseases using B-mode ultrasound images.

**Methods:**

This research utilizes 2064 B-mode ultrasound images of the eye to train a novel model integrating artificial intelligence technology.

**Results:**

The ConvNeXt-L model achieved outstanding performance with an accuracy rate of 84.3% and a Kappa value of 80.3%. Across five classifications (no obvious abnormality, vitreous opacity, posterior vitreous detachment, retinal detachment, and choroidal detachment), the model demonstrated sensitivity values of 93.2%, 67.6%, 86.1%, 89.4%, and 81.4%, respectively, and specificity values ranging from 94.6% to 98.1%. F1 scores ranged from 71% to 92%, while AUC values ranged from 89.7% to 97.8%.

**Conclusion:**

Among various models compared, the ConvNeXt-L model exhibited superior performance. It effectively categorizes and visualizes pathological changes, providing essential assisted information for ophthalmologists and enhancing diagnostic accuracy and efficiency.

## 1 Introduction

Eye B-mode ultrasonography is a prevalent medical classification approach employed to evaluate eye structures and identify eye diseases. This technique is particularly valuable for examining intraocular diseases, especially when obstructions like refractive media opacity or lesions situated within the orbit are present (Bates and Goett, 2022). Leveraging ultrasound technology, it produces real-time images of internal eye structures and holds extensive clinical utility. Diseases such as vitreous opacities, posterior vitreous detachment (PVD), retinal detachment, and choroidal detachment represent common eye ailments (Bellows et al., 1981; Ghazi and Green, 2002; Shinar et al., 2011; Gishti et al., 2019; Ryan, 2021), often discernible through eye B-mode ultrasound images.

Eye B-mode ultrasound image classification traditionally relies on the expertise of ophthalmologists. However, an uneven distribution of medical professionals across different regions in China presents a challenge (Jiao and Wang, 2022). The majority of ophthalmologists are concentrated in economically developed eastern coastal areas. Consequently, eye B-mode ultrasonography is often conducted by medical imaging specialists who may lack the specialized knowledge required for accurate interpretation. As a result, misdiagnoses occur due to the absence of proficiency in ocular ultrasonography interpretation. This underscores the current shortage of skilled professionals capable of effectively analyzing B-mode ultrasound images, particularly in numerous grassroots hospitals, leading to an inability to fulfill the demand for precise image analysis.

Recently, the integration of artificial intelligence (AI) into the medical domain has emerged as a prominent research area. Notably, machine learning and deep learning techniques have demonstrated substantial efficacy in medical diagnostics. Machine learning, characterized by the use of manually selected features and algorithms for disease identification, has yielded satisfactory outcomes (Rajan and Ramesh, 2015; Chowdhury and Banerjee, 2016; Hosoda et al., 2020; Xu et al., 2020; Kooner et al., 2022; Yang et al., 2023). On the other hand, deep learning harnesses convolutional neural networks to automatically extract features from images (Mirzania et al., 2021; Ho et al., 2022; Xie et al., 2022; Chen et al., 2023; Li et al., 2023). In a study by Nagasato et al., a comparison between machine learning and deep learning in branch retinal vein occlusion (RVO) detection using ultra-widefield retinal images revealed that deep learning exhibited superior sensitivity and specificity (Nagasato et al., 2019). This trend has consequently spurred numerous researchers to leverage deep learning methodologies for the diagnosis of eye diseases.

For instance, Zhu et al. harnessed machine learning to predict changes in spherical equivalent refraction (SER) and axial length (AL) in children, yielding impressive R2 values of 89.97% for SER and 75.46% for AL prediction (Zhu et al., 2023). Employing a deep learning model, Li et al. successfully discriminated between normal fundus images and eight prevalent eye diseases, attaining an exceptional area under the receiver operating characteristic curve (AUC) of 97.84% (Li et al., 2020). In their work, He et al. proposed a binary classification and segmentation model tailored for pterygium diagnosis, achieving a remarkable classification accuracy of 99% (Zhu et al., 2022). Meanwhile, Chen et al. devised a deep learning system for swift screening of rhegmatogenous retinal detachment (RRD), vitreous detachment (VD), and vitreous hemorrhage (VH), culminating in accuracies of 94, 90, 92, 94, and 0.91% for normal, VD, VH, RD, and other lesions, respectively (Chen et al., 2021). Notably, Zheng et al. crafted a common retinal disease classification model grounded in ResNet50 architecture using a dataset of 2000 fundus images, effectively diagnosing various eye diseases except macular degeneration (MD) (Zheng et al., 2021). Moreover, Google’s team, led by Gulshan et al., skillfully trained a deep learning model to diagnose diabetic retinopathy (DR) and assign a severity grade through fundus images. Their efforts culminated in compelling outcomes validated through clinical trials (Ahn et al., 2018).

To tackle the challenge of intelligent assistance in the analysis of eye disease B-mode ultrasound images, this study leverages the ConvNeXt-L model (Liu et al., 2022) to train a intelligence-assisted five-classification framework for common eye diseases. This is the first application of the ConvNeXt model in Eye B-mode ultrasound image classification for eye diseases. This framework aids non-specialist ophthalmologists in conducting preliminary patient diagnoses, facilitating the identification of no significant abnormalities and four common eye diseases (vitreous opacities, posterior vitreous detachment, retinal detachment, choroidal detachment), while also conducting visualization analysis. This innovative approach seeks to bridge the gap between the substantial patient volume encountered in grassroots hospitals and the limited accessibility of specialized ophthalmologists. By pursuing this approach, the objective is to deliver efficacious services to individuals with eye diseases. The subsequent findings are delineated as follows.

## 2 Materials and methods

### 2.1 Data source

This study encompassed experiments conducted on a dataset of common eye disease B-mode ultrasound images, generously contributed by collaborative hospitals. The research conformed to the ethical principles outlined in the Helsinki Declaration. To ensure data providers’privacy, the study did not impose any restrictions on patients’age or gender within the images. A rigorous anonymization process was undertaken, eliminating all personally identifiable information to uphold patient confidentiality. Consequently, no patient demographics are available. The research incorporated a total of 2064 images, with 1860 allocated for the training set and 204 for the test set, as detailed in Table 1.

**Table 1:** Eye disease B-mode ultrasound image dataset.

Category	Training dataset	Test dataset
Normal	407	44
Vitreous opacities	291	36
Posterior vitreous detachment	351	34
Retinal detachment	427	47
Choroidal detachment	384	43
Total	1860	204

### 2.2 ConvNeXt model

This investigation utilized the ConvNeXt-L model, which was pre-trained on the ImageNet Large Scale Visual Recognition Challenge (ILSVRC) dataset. Leveraging a dataset comprising 1860 images, encompassing “No Abnormalities” images alongside four common eye disease B-mode ultrasound images, a five-class intelligence-assisted diagnostic model for prevalent eye diseases was meticulously trained. This adeptly trained model exhibits proficiency in classifying both “No Abnormalities” images and the four prevalent eye diseases. Furthermore, it employs visualization techniques to unveil focal regions within the images, contributing to a comprehensive diagnostic process.

This study incorporated additional well-established deep learning classification models, namely ResNet50 (Targ et al., 2016) and EfficientNet-B4 (Tan and Le, 2019). These models principally comprise convolutional layers, pooling layers, and fully connected layers. The ConvNeXt model, a refined iteration of the ResNet-50 model, is available in distinct versions like ConvNeXt-T, ConvNeXt-S, ConvNeXt-B, ConvNeXt-L, and ConvNeXt-XL. Notably, these models differ primarily in their network structure’s depth and width.

**Figure 1:**
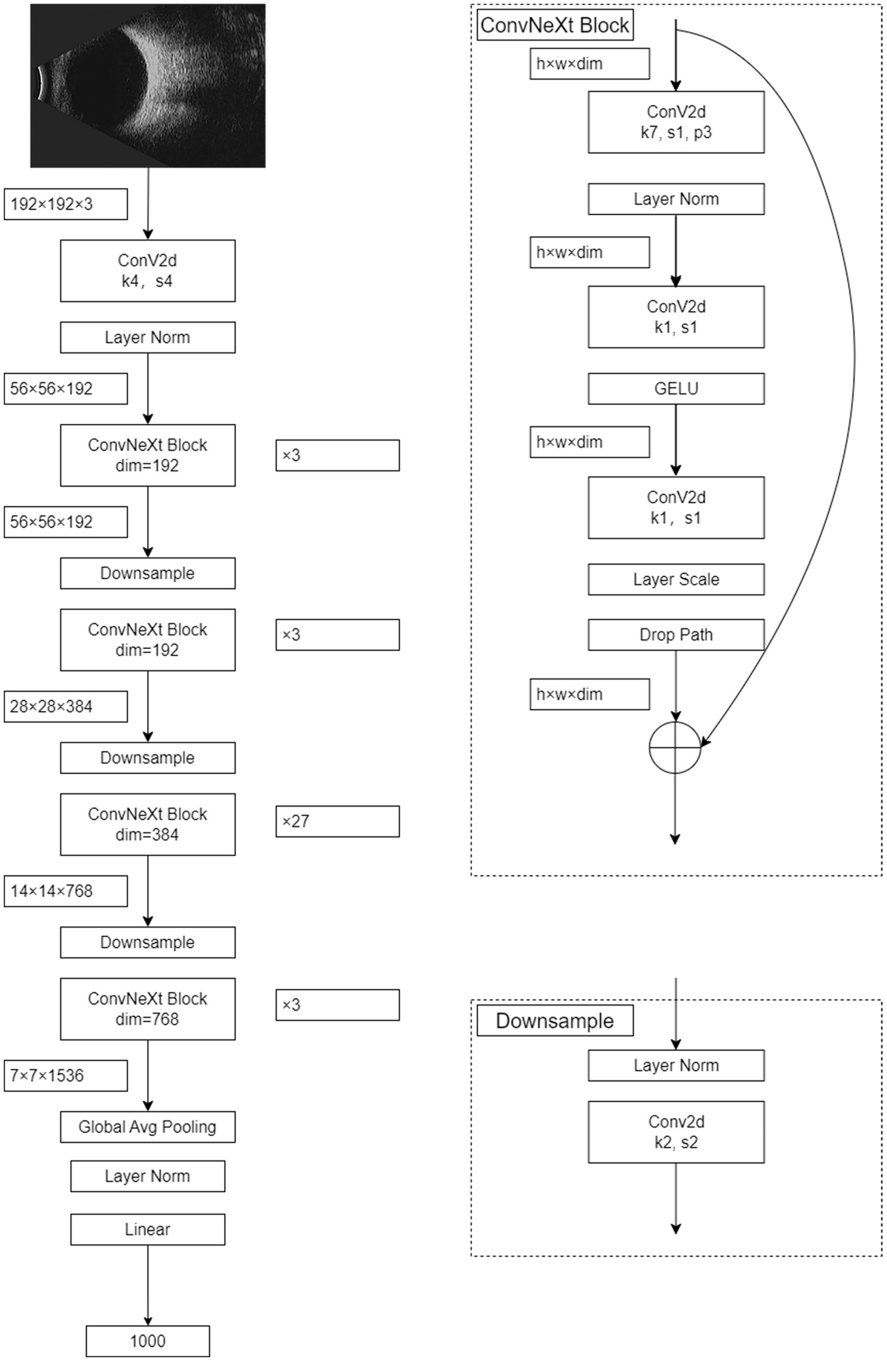
The structure of the ConvNeXt model.

The foundational structure of the ConvNeXt model encompasses convolutional layers, activation layers, and fully connected layers. The architecture of the ConvNeXt-L model integrates a 4×4 convolutional layer alongside 36 ConvNeXtBlocks of varying sizes. Each ConvNeXtBlock comprises three convolutional layers of different dimensions, along with LayerNorm, GELU activation, LayerScale, and DropPath components. For ConvNeXt-L, input images undergo preprocessing and resizing to 192×192 dimensions, derived from eye B-mode ultrasound images, culminating in the final five-class intelligent diagnostic model. Figure 1 visually outlines the architecture of the ConvNeXt-L model.

To enhance the precision of eye disease classification in B-mode ultrasonography, this study conducted a comprehensive model comparison. The examined models encompassed ResNet50, EfficientNet-B4, DeiT3 (Touvron et al., 2022), and Swin Transformer V2 (Liu et al., 2022). Notably, ResNet50 and EfficientNet-B4 belong to the domain of deep convolutional neural networks, while DeiT3 and Swin Transformer V2 adopt attention-based neural network architectures. These four models were subjected to experimentation using the eye B-mode ultrasound image dataset. Subsequently, the most superior-performing model was meticulously identified for the accurate classification of eye B-mode ultrasonography diseases.

### 2.3 Statistical analysis

Statistical analysis was performed using SPSS 27.0 software. Firstly, count data was presented in the form of both image numbers and percentages. Subsequently, common metrics for diagnosing eye diseases, such as sensitivity, specificity, and F1 score, were calculated for both normal fundus images and images associated with four common diseases. Following this, the receiver operating characteristic (ROC) curve was plotted to evaluate the model’s performance. Additionally, we employed the kappa statistic to assess the agreement between the diagnoses made by expert clinicians and the model’s diagnostic results. In this context, the results from the expert clinician group were taken as the ground truth, and the kappa statistic was utilized to quantify the level of agreement. Typically, a kappa value between 0.61 and 0.80 indicates substantial agreement, while a kappa value exceeding 0.80 suggests high agreement. Through the aforementioned analyses, conclusions regarding the performance and consistency of the eye disease diagnostic model can be drawn.

## 3 Results

### 3.1 Expert classification results

The study employed 1860 eye B-mode ultrasound images for training and 204 images for testing. The image collection format remained consistent throughout. Among the entire set of eye B-mode ultrasound images, expert clinicians identified 451 as normal, 327 as vitreous opacity, 385 as posterior vitreous detachment, 474 as retinal detachment, and 427 as choroidal detachment.

### 3.2 Intelligent-assisted classification results

The confusion matrices for the diagnostic results of the ResNet50, EfficientNet-B4, DeiT3, Swin Transformer V2, and ConvNeXt-L models can be found in Tables 2–6.

**Table 2:** ResNet50 model classification results.

	Normal	Vitreous opacities	Posterior vitreous detachment	Retinal detachment	Choroidal detachment
Normal	41	0	3	0	0
Vitreous opacities	0	25	6	3	2
Posterior vitreous detachment	3	8	23	0	0
Retinal detachment	0	0	2	39	6
Choroidal detachment	0	0	1	9	33

**Table 3:** EfficientNet-B4 model classification results.

	Normal	Vitreous opacities	Posterior vitreous detachment	Retina ldetachment	Choroidal detachment
Normal	42	0	2	0	0
Vitreous opacities	0	25	4	3	4
Posterior vitreous detachment	4	12	17	0	1
Retinal detachment	0	0	2	41	4
Choroidal detachment	0	3	0	5	35

**Table 4:** DeiT3 model classification results.

	Normal	Vitreous opacities	Posterior vitreous detachment	Retina ldetachment	Choroidal detachment
Normal	42	0	2	0	0
Vitreous opacities	1	30	4	1	0
Posterior vitreous detachment	4	10	20	0	0
Retinal detachment	0	1	0	42	4
Choroidal detachment	0	1	0	5	37

**Table 5:** Swin transformer V2 model classification results.

	Normal	Vitreous opacities	Posterior vitreous detachment	Retina ldetachment	Choroidal detachment
Normal	42	0	2	0	0
Vitreous opacities	0	26	5	5	0
Posterior vitreous detachment	3	14	16	0	1
Retinal detachment	0	0	1	43	3
Choroidal detachment	0	2	0	8	33

**Table 6:** ConvNeXt-L model classification results.

	Normal	Vitreous opacities	Posterior vitreous detachment	Retina ldetachment	Choroidal detachment
Normal	41	0	3	0	0
Vitreous opacities	0	31	3	1	1
Posterior vitreous detachment	4	7	23	0	0
Retinal detachment	0	1	2	42	2
Choroidal detachment	0	1	0	7	35

### 3.3 Evaluation metric results

The evaluation metric results for the five models: ResNet50, EfficientNet-B4, DeiT3, Swin Transformer V2, and ConvNeXt-L, are presented in Table 7.

**Table 7:** Evaluation metric results for the five models.

Model	Evaluation metrics	Normal	Vitreous opacities	Posterior vitreous detachment	Retina ldetachment	Choroidal detachment
ResNet50	Sensitivity	93.2%	67.6%	69.4%	83.0%	76.7%
	Specificity	98.1%	92.9%	95.2%	92.4%	95.0%
	F1Score	93%	67%	72%	80%	79%
	AUC	98.6%	88.9%	91.7%	97.4%	96.7%
	Kappa	73.5%				
	ACC	78.9%				
EfficientNet-B4	Sensitivity	95.5%	50.0%	69.4%	87.2%	81.4%
	Specificity	98.1%	92.9%	95.0%	92.4%	95.0%
	F1Score	93%	67%	72%	80%	79%
	AUC	98.6%	88.9%	91.7%	97.4%	96.7%
	Kappa	73.5%				
	ACC	78.9%				
DeiT3	Sensitivity	95.5%	58.8%	83.3%	98.0%	97.5%
	Specificity	96.9%	96.5%	92.9%	96.2%	97.5%
	F1Score	92%	67%	77%	88%	88%
	AUC	99.8%	87.5%	94.8%	98.0%	97.5%
	Kappa	79.7%				
	ACC	83.8%				
Swin transformer V2	Sensitivity	95.5%	47.1%	72.2%	91.5%	76.7%
	Specificity	98.1%	95.3%	90.5%	91.7%	97.5%
	F1Score	94%	55%	67%	83%	82%
	AUC	99.7%	92.4%	95.1%	98.2%	98.3%
	Kappa	72.9%				
	ACC	78.4				
ConvNeXt-L	Sensitivity	93.2%	67.6%	86.1%	89.4%	81.4%
	Specificity	97.5%	95.3%	94.6%	94.9%	98.1%
	F1 Score	92%	71%	82%	87%	86%
	AUC	95.4%	89.7%	95.0%	97.1%	97.8%
	Kappa	80.3%				
	ACC	84.3%				

### 3.4 Visualization study

**Figure 2:**
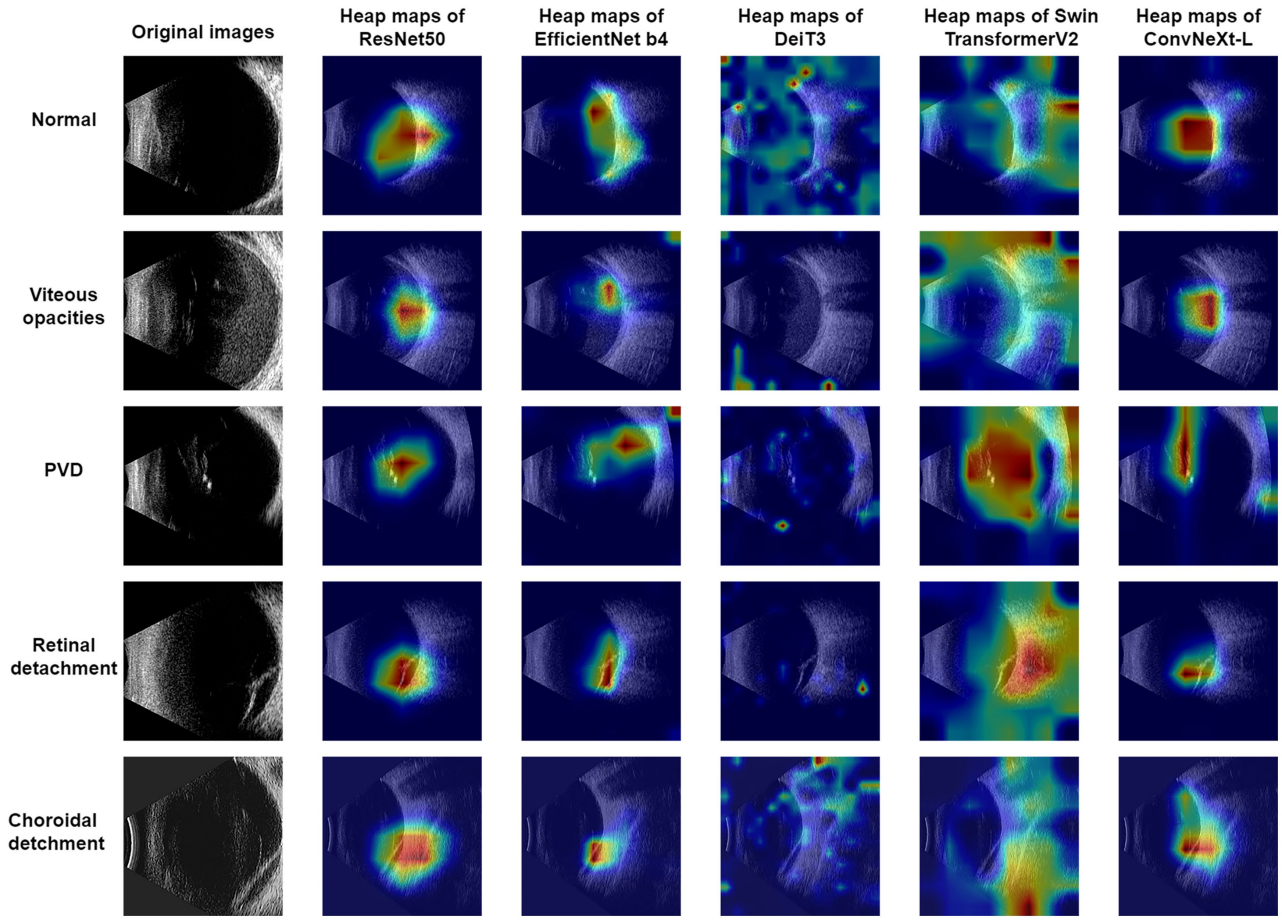
B-mode ultrasound images and four common eye diseases, along with visualization results.

In this research, Grad-CAM (Selvaraju et al., 2017) was harnessed to produce visualizations for the ResNet50, EfficientNet-B4, DeiT3, Swin Transformer V2, and ConvNeXt-L models, as showcased in Figure 2. An observation from the images reveals that ConvNeXt-L’s visualization showcases the most accurate annotated regions. In comparison, the annotated regions in the visualizations for the remaining four models exhibit a slightly lower precision. It’s noteworthy that these visualizations for the five models are generated through the same algorithm. Consequently, as the overall performance of the models advances, the accuracy of the annotated regions in the visualizations proportionally improves.

## 4 Discussion

Eye ultrasonography stands as a pivotal imaging technique for evaluating eye and adjacent tissue structures (Elabjer et al., 2017). In the realm of ophthalmology, it holds a prominent status as a diagnostic imaging approach, offering a secure, non-invasive avenue with instantaneous, real-time feedback. Its integration into ophthalmic evaluation traces back to the 1950s, spurred by the accessibility of eye anatomy (Bangal et al., 2016). Notably, the eye’s cystic nature renders it an ideal candidate for ultrasound examination facilitated by high-frequency sound waves (Uduma et al., 2019). The merits of eye ultrasound examination lie in its attributes of non-invasiveness, cost-effectiveness, real-time functionality, and the potential for repetition in diagnosing eye pathologies. As a consequence, eye ultrasound examination has evolved into an indispensable tool, effectively serving the diagnosis and treatment of a spectrum of ophthalmic diseases.

As scientific and technological progress continues, the landscape of artificial intelligence (AI) is undergoing rapid transformation, venturing into uncharted territories. An illustrative example is the landmark introduction of AlexNet by Krizhevsky et al. (2012) and colleagues, which remarkably outperformed competitors in the ImageNet competition. This breakthrough ignited widespread fascination with neural networks, laying the groundwork for tackling image-related challenges using deep neural networks. This catalyst subsequently led to a proliferation of exceptional accomplishments in the realm. In 2015, the introduction of ResNet (Simonyan and Zisserman, 2014), pioneered by Kaiming He and team, secured a triumph in the ImageNet competition, pushing image classification error rates to a remarkable low of 3.6%, even surpassing human recognition capacities.

Further fueling progress, Google’s application of the Transformer to image classification tasks in 2020 birthed the revolutionary Vision Transformer (ViT) model (Dosovitskiy et al., 2020). Another milestone occurred in 2022, with the unveiling of the ConvNeXt model by Liu et al. (2022). This innovation drew inspiration from the Swin Transformer architecture, achieving advancements by refining ResNet50. The ConvNeXt-L variant notably achieved heightened performance, boasting an accuracy of 87.8% on ImgNet 22 k. The pervasive influence of AI extends across diverse domains, with the medical sphere being no exception. Noteworthy instances include Zheng et al. (2021), who harnessed 2000 fundus images to devise a model employing ResNet50 for classifying common retinal diseases, and Zhu et al. (2022), who effectively utilized the EfficientNet-B4 model for diagnosing a range of retinal diseases, yielding promising results.

This study employed five models for experimentation: ResNet50, EfficientNet-B4, DeiT3, Swin Transformer V2, and ConvNeXt-L. Among them, the ConvNeXt-L model exhibited the best classification performance. Several factors could contribute to its superior performance over the other four models. Firstly, the ConvNeXt-L model ingeniously tweaks the ResNet50 architecture, infusing it with design principles inspired by Transformers while retaining convolutional layers instead of attention modules. This strategic fusion enhances the ConvNeXt-L model’s feature extraction capabilities, amplifying its strength in classification. Secondly, the ConvNeXt-L model underwent training on the ImgNet 22 k dataset, leveraging a pretrained model that was subsequently fine-tuned in this investigation. The model’s exposure to a diverse array of images during its pretraining phase ostensibly influenced its performance boost. The ConvNeXt model encompasses an assortment of five versions, namely ConvNeXt-T, ConvNeXt-S, ConvNeXt-B, ConvNeXt-L, and ConvNeXt-XL. The elemental divergence across these versions pertains to the depth and breadth of their network structures. Importantly, ConvNeXt-L distinguishes itself with its enhanced feature extraction prowess and a relatively streamlined parameter count, culminating in expedited execution speeds compared to the remaining four variants.

Using the Grad-CAM method, this study produced visualizations. From Figure 2, it can be observed that the ConvNeXt-T model exhibited the most optimal performance, with its annotated regions closely aligning with those identified by ophthalmologists. Among the remaining four models, apart from the DeiT3 model, which exhibited a substantial disparity between its visualized annotations and the ophthalmologists’ diagnostic regions, the performances were relatively comparable to the ConvNeXt-L model’s results. DeiT3’s classification performance was not poor; however, the poor visualized annotations might be attributed to a misalignment with the Grad-CAM methodology.

In Table 7, the models demonstrated relatively higher sensitivity and specificity scores for the diseases of Normal, Retinal Detachment, and Choroidal Detachment. However, for the diseases of Vitreous Opacity and Posterior Vitreous Detachment, the sensitivity and specificity scores were notably lower. This discrepancy arises from the models difficulty in distinguishing between Vitreous Opacity and Posterior Vitreous Detachment, likely due to the striking similarity of pathological regions in these two diseases on eye B-mode ultrasound images. The low sensitivity and specificity scores for these two diseases can be attributed to their confusion by the model, which could potentially be mitigated by an increased dataset size.

Tables 1–6 collectively demonstrate that these five models are capable of diagnosing Normal and the other four common eye diseases. However, errors are more prone to occur in the classification of the four common eye diseases, particularly Posterior Vitreous Detachment versus Vitreous Opacity, and Retinal Detachment versus Choroidal Detachment. Therefore, when the model diagnoses any of the four diseases apart from Normal, it is recommended for physicians to reconsider the diagnosis or for patients to seek further evaluation at a higher-level medical institution to prevent misdiagnosis or missed diagnosis.

Although, this research does have some limitations. Primarily, the model faces challenges in diagnosing detachment of the vitreous from the retina and vitreous opacities, as well as the immature application of transfer learning on medical imaging datasets. Additionally, there is still room for improvement in the model’s interpretability. The model frequently confuses Posterior Vitreous Detachment with Vitreous Opacity, and Choroidal Detachment with Retinal Detachment. Thus, the five-classification model based on ConvNeXt-L is only suitable for preliminary diagnosis of common eye diseases. This issue might stem from the fact that a single eye B-mode ultrasound image can have multiple labels, while the model provides only the most severe diagnosis, leading to incorrect results. The accuracy of the model in diagnosing Normal and the four common eye diseases ranges from 78 to 84%, indicating that the accuracy is not exceptionally high. The slightly lower accuracy is mainly due to the limited dataset and the presence of multiple labels per image. To elevate accuracy, specificity, and sensitivity, strategies like data augmentation, employing Generative Adversarial Networks (GANs) for image generation, and expanding the dataset’s scope could be harnessed. Due to the incomplete maturity of transfer learning in medical imaging datasets, we will further explore its potential on such datasets to enhance the classification accuracy of eye diseases. Meanwhile, considering the limitations of model interpretability, we plan to adopt ensemble methods to delve into the crucial features in the model decision-making process, aiming to further improve model interpretability.

## 5 Conclusion

In conclusion, this study proposes an intelligence-assisted five-classification model for common eye diseases B-mode ultrasound images, focusing on four common eye diseases. The model aims to intelligently analyze and visualize images of these four common eye diseases. This study employed the ConvNeXt-L model to design an intelligence-assisted five-classification model for common eye diseases in eye B-mode ultrasound images. This model contributes to the enhancement of classification accuracy and timeliness for common eye diseases in underdeveloped regions. In the future, this model will be refined to provide more comprehensive assisted classification results for patients with eye diseases, thus covering an even broader range of eye diseases and adaptable to various scenarios.

## Data availability statement

The original contributions presented in the study are included in the article/supplementary material, further inquiries can be directed to the corresponding authors.

## Author contributions

SZ: Writing – original draft, Writing – review & editing. XL: Writing – review & editing, Writing – original draft. YL: Writing – review & editing. BZ: Writing – review & editing. MW: Writing – review & editing. XY: Writing – review & editing, Conceptualization, Data curation, Methodology. WY: Writing – review & editing, Conceptualization, Funding acquisition, Methodology. YG: Conceptualization, Funding acquisition, Methodology, Writing – review & editing.
